# Supplementary material for: Genetic Diversity of Leuconostoc mesenteroides Isolates from Traditional Montenegrin Brine Cheese
Source: Microorganisms. 2021 Jul 28;9(8):1612. doi: 10.3390/microorganisms9081612 (PMC8401054; doi:10.3390/microorganisms9081612)

Figure S1. Regions of Montenegro with respect to climate, environmental conditions and regional food specialities. Locations of cheese producers marked in green. Adapted from Martinović et al. 2021 [52].

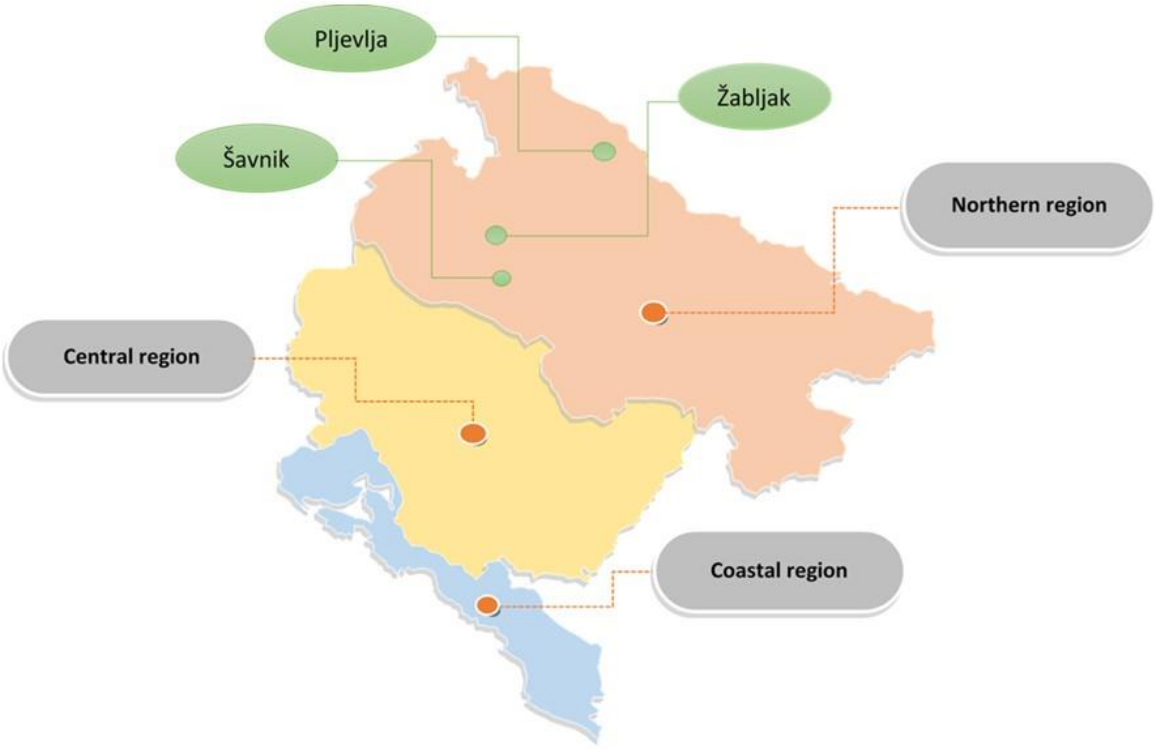

Supplement: Supplementary file 1 [file microorganisms-09-01612-s001.zip › Supplementary Figure S1 Regions of Montenegro.pdf]
