# Supplementary material for: Genetic Diversity of Leuconostoc mesenteroides Isolates from Traditional Montenegrin Brine Cheese
Source: Microorganisms. 2021 Jul 28;9(8):1612. doi: 10.3390/microorganisms9081612 (PMC8401054; doi:10.3390/microorganisms9081612)

Figure S4. MLST: Minimum spanning tree (MST) comprising the 16 Montenegrin brine cheese *Ln. mesenteroides* isolates and 72 isolates downloaded from GenBank. Isolates are colored according to their country of origin.

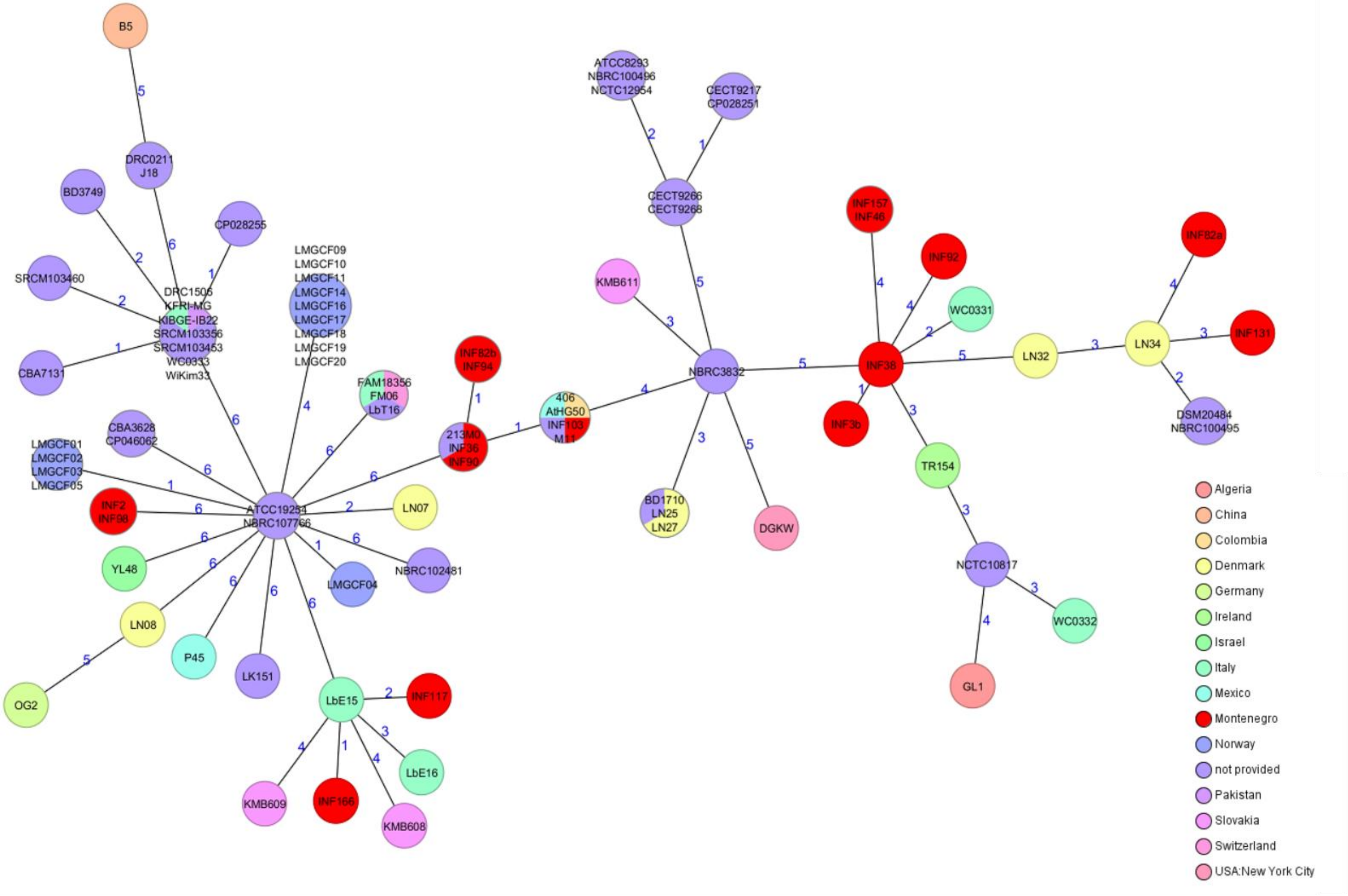

Supplement: Supplementary file 1 [file microorganisms-09-01612-s001.zip › Supplementary Figure S4 MLST.pdf]
