# Supplementary material for: Genetic Diversity of Leuconostoc mesenteroides Isolates from Traditional Montenegrin Brine Cheese
Source: Microorganisms. 2021 Jul 28;9(8):1612. doi: 10.3390/microorganisms9081612 (PMC8401054; doi:10.3390/microorganisms9081612)
Supplement: Supplementary file 1 [file microorganisms-09-01612-s001.zip › Supplementary Table S1_cgMLST_scheme_data.pdf]

Seed Genome:

* NC_008531.1 (25-AUG-2019), 2038396 bases, 1955 genes with CDS (Leuconostoc mesenteroides subsp. mesenteroides ATCC 8293, complete genome)

Penetration Query Genomes (18):

* NC_016805.3 (11-FEB-2020), 1900740 bases, 1795 genes with CDS (Leuconostoc mesenteroides subsp. mesenteroides J18, complete sequence)

* NZ_CP000574.1 (29-NOV-2019), 1896413 bases, 1820 genes with CDS (Leuconostoc mesenteroides KFRI-MG, complete genome)

* NZ_CP012009.1 (01-DEC-2019), 1818633 bases, 1784 genes with CDS (Leuconostoc mesenteroides subsp. dextranicum strain DSM 20484, complete genome)

* NZ_CP014610.1 (05-DEC-2019), 1986568 bases, 1925 genes with CDS (Leuconostoc mesenteroides subsp. mesenteroides strain BD3749, complete genome)

* NZ_CP014611.1 (19-FEB-2020), 1893478 bases, 1805 genes with CDS (Leuconostoc mesenteroides subsp. jonggajibkimchii strain DRC1506 chromosome, complete genome)

* NZ_CP013016.1 (17-OCT-2019), 1923948 bases, 1828 genes with CDS (Leuconostoc mesenteroides subsp. mesenteroides strain DRC0211, complete genome)

* NZ_CP015442.1 (17-DEC-2019), 2124039 bases, 2057 genes with CDS (Leuconostoc mesenteroides subsp. mesenteroides strain BD1710 chromosome, complete genome)

* NZ_CP020731.1 (20-DEC-2019), 1891719 bases, 1871 genes with CDS (Leuconostoc mesenteroides subsp. mesenteroides FM06, complete genome)

* NZ_AP017936.1 (21-DEC-2019), 2090103 bases, 2025 genes with CDS (Leuconostoc mesenteroides DNA, complete genome, strain: LK-151)

* NZ_CP021491.1 (08-MAR-2020), 1894793 bases, 1833 genes with CDS (Leuconostoc mesenteroides strain WiKim33 chromosome, complete genome)

* NZ_CP021966.1 (19-FEB-2020), 2004569 bases, 1945 genes with CDS (Leuconostoc mesenteroides strain CBA7131 chromosome, complete genome)

* NZ_CP035139.1 (06-MAR-2020), 1946833 bases, 1911 genes with CDS (Leuconostoc mesenteroides strain SRCM103356 chromosome, complete genome)

* NZ_CP035271.1 (06-MAR-2020), 2057400 bases, 2027 genes with CDS (Leuconostoc mesenteroides strain SRCM103453 chromosome, complete genome)

* NZ_CP035746.1 (09-MAR-2020), 1980926 bases, 1934 genes with CDS (Leuconostoc mesenteroides strain SRCM103460 chromosome, complete genome)

* NZ_CP042404.1 (16-AUG-2019), 1777320 bases, 1692 genes with CDS (Leuconostoc mesenteroides strain CBA3628 chromosome, complete genome)

* NZ_CP046062.1 (22-NOV-2019), 1777316 bases, 1687 genes with CDS (Leuconostoc mesenteroides subsp. mesenteroides strain CBA3607 chromosome, complete genome)

* NZ_CP028251.1 (23-JAN-2020), 1988249 bases, 1900 genes with CDS (Leuconostoc mesenteroides strain SRCM102733 chromosome, complete genome)

* NZ_CP028255.1 (23-JAN-2020), 1973483 bases, 1905 genes with CDS (Leuconostoc mesenteroides strain SRCM102735 chromosome, complete genome)

Seed Genome Filters:

* Minimum Length Filter (requires >=50 bases)

* Start Codon Filter (requires start codon at beginning of the gene)

* Stop Codon Filter (requires single stop codon at end of gene)

* Homologous Gene Filter (requires no multiple copies of gene with BLAST overlap>=100bp, identity>=90.0%)

* Gene Overlap Filter (requires no overlap with other genes >4 bases)

Query Genome BLAST Search:

* Requires BLAST hit with overlap=100%, identity>=90.0% in every query genome

* BLAST options: Word size=11, Mismatch penalty=-1, Match reward=1, Gap open costs=5, Gap extension costs=2

Penetration Query Genomes Filters:

* Stop Codon Percentage Filter (requires single stop codon at end of gene in >80% penetration query genomes)

BLAST version 2.2.12

Citation: Altschul SF, Gish W, Miller W, Myers EW, Lipman DJ. 1990. Basic local alignment search tool. J Mol Biol 215:403-410.
