# Supplementary material for: Genetic Diversity of Leuconostoc mesenteroides Isolates from Traditional Montenegrin Brine Cheese
Source: Microorganisms. 2021 Jul 28;9(8):1612. doi: 10.3390/microorganisms9081612 (PMC8401054; doi:10.3390/microorganisms9081612)
Supplement: Supplementary file 1 [file microorganisms-09-01612-s001.zip › Supplementary Table S4_genome_data.pdf]

Table S4. Genome data. Lowest allelic distance of Montenegrin brine cheese isolates to reference strains are indicated in green color. StrainID colors indicate strains differentiated by less than 45 alleles in their core genome. (P) Producers.

P	Cheese ID	Strain ID	Subsp.-Id<br>TYGS	Allelic distance ATCC8293	Allelic distance ATCC19254	Allelic distance DSM20484	Allelic distance DRC1506	Coverage	Contigs	Genome size (Mb)	Genes	Coding genes	Pseudo Genes	RNA Genes	Accession No.
**A**	2-1406	INF2	*Ln. m. cremoris?*	844	790	811	810	41	192	2.03	2,202	1,992	150	60	JAGFYL000000000
	3-1406	INF3b	*Ln. m. mesenteroides*	793	814	742	816	45	233	2.13	2,231	2,121	110	61	JAFREC000000000
**B**	1-1404	INF36	*Ln. m. mesenteroides*	635	811	863	805	51	246	2.16	2,232	2,141	91	65	JAFREB000000000
	2-1404	INF38	*Ln. m. mesenteroides*	781	814	767	805	37	163	2.06	2,173	2,011	99	63	JAFREA000000000
**C**	1453	INF46	*Ln. m. mesenteroides*	793	820	792	816	31	205	2.04	2,153	1,979	109	65	JAFRDZ000000000
**D**	1-3763	INF82a	*Ln. m. dextranicum*	849	792	293	818	26	351	2.14	2,295	2.094	139	62	JAFRDY000000000
		INF82b	*Ln. m. mesenteroides*	637	815	865	809	40	184	2.25	2,365	2,205	100	60	JAGFYK000000000
	3-3763	INF90	*Ln m. mesenteroides*	634	815	863	809	41	258	2.23	2,368	2,221	85	62	JAFRDX000000000
		INF92	*Ln. m. mesenteroides*	791	813	755	815	21	233	1.99	2,074	1,905	109	60	JAFRDW000000000
		INF94	*Ln. m. mesenteroides*	621	803	849	793	29	388	2.18	2,292	2,144	89	59	JAFRDV000000000
	4-3763	INF98	*Ln. m. cremoris?*	841	787	809	808	32	313	2.02	2,169	1,958	151	60	JAFRDU000000000
**E**	4-3758	INF103	*Ln. m. mesenteroides*	576	765	800	754	10	515	2.10	2,259	2,107	95	57	JAFRDT000000000
**F**	4-14246	INF117	*Ln. m. jonggajibkimchii?*	786	811	861	810	25	288	2.02	2,183	2,022	102	59	JAGFYJ000000000
**G**	Ž	INF 131	*Ln. m. dextranicum*	699	797	358	824	25	216	2.03	2,139	1,958	121	60	JAGFYI000000000
**H**	3-1387*	INF157	*Ln. m. mesenteroides*	793	818	791	816	93	91	1.99	2,010	1,888	84	38	JAGSYL000000000
**I**	4-1383*	INF166	*Ln. m. jonggajibkimchii?*	774	811	850	807	39	113	2.03	2,185	2,018	107	60	JAFRDS000000000
